# Impact of Setmelanotide on Metabolic Syndrome Risk in Patients With Bardet-Biedl Syndrome

**DOI:** 10.1210/clinem/dgaf079

**Published:** 2025-02-07

**Authors:** Andrea M Haqq, Christine Poitou, Wendy K Chung, Elizabeth Forsythe, Rushika Conroy, Hélène Dollfus, Sonali Malhotra, Nicolas Touchot, Uzoma Okorie, Philip Beales, Karine Clément, Jesús Argente

**Affiliations:** Division of Pediatric Endocrinology, University of Alberta, Edmonton, AB, Canada T6G 2B7; Nutrition Department, Reference Center of Rare Diseases PRADORT, Assistance Publique Hôpitaux de Paris, Pitié-Salpêtrière Hospital, Paris 75013, France; Sorbonne Université, Inserm, Nutrition and Obesities, Systemic Approaches NutriOmique Research Group, Paris 75013, France; Department of Pediatrics, Boston Children's Hospital, Harvard Medical School, Boston, MA 02115, USA; Clinical Genetics Department, Guys and St. Thomas’ Hospitals, London; National Bardet-Biedl Syndrome Clinics, Great Ormond Street Hospital, London WC1N 3BH, UK; Division of Weight Management, Division of Pediatric Endocrinology, Maine Medical Center, Portland, ME 04102, USA; CARGO and Department of Medical Genetics, Hôpitaux Universitaires de Strasbourg, Institut de Génétique Médicale d’Alsace, Université de Strasbourg, Strasbourg 67000, France; Rhythm Pharmaceuticals, Inc., Boston, MA 02116, USA; Massachusetts General Hospital, Boston, MA 02114, USA; Rhythm Pharmaceuticals, Inc., Boston, MA 02116, USA; Marshfield Clinic Research Institute, Marshfield, WI 54449, USA; Genetics and Genomic Medicine Programme, University College London Great Ormond Street Institute of Child Health, London WC1N 1EH, UK; Nutrition Department, Reference Center of Rare Diseases PRADORT, Assistance Publique Hôpitaux de Paris, Pitié-Salpêtrière Hospital, Paris 75013, France; Sorbonne Université, Inserm, Nutrition and Obesities, Systemic Approaches NutriOmique Research Group, Paris 75013, France; Department of Pediatrics and Pediatric Endocrinology, Universidad Autónoma de Madrid, University Hospital Niño Jesús, Madrid 28009, Spain; CIBER “Fisiopatología de la obesidad y nutrición” (CIBEROBN), Instituto de Salud Carlos III, Madrid 28029, Spain; IMDEA Food Institute, Madrid 28049, Spain

**Keywords:** cardiovascular disease, type 2 diabetes, genetics/genomics, comorbidities, metabolic syndrome, hypothalamus

## Abstract

**Context:**

Bardet-Biedl syndrome (BBS) is a rare genetic disease associated with disruptions in melanocortin-4 receptor pathway signaling that can contribute to increased risk for metabolic syndrome and obesity-related comorbidities.

**Objective:**

Here, MetS-Z-BMI scores, a continuous measure based on body mass index (BMI), were calculated to determine metabolic syndrome severity and response to treatment with the melanocortin-4 receptor agonist setmelanotide in BBS.

**Methods:**

All patients from a phase 3 study (NCT03746522) of setmelanotide with data required for the calculation of MetS-Z-BMI scores were included. Mean MetS-Z-BMI score was determined at baseline and Week 52; subgroup analyses were conducted by sex, age, genotype, and response to setmelanotide.

**Results:**

MetS-Z-BMI scores were evaluable for 22 of 32 patients enrolled. Baseline mean (SD) MetS-Z-BMI score across patients was 1.1 (0.5); baseline mean (SD) odds ratios of future cardiovascular disease or type 2 diabetes were 3.1 (1.5) and 3.7 (1.7), respectively, for adults and 10.2 (4.7) and 2.8 (1.3), respectively, for pediatric patients. Overall, mean (SD) MetS-Z-BMI score at Week 52 was reduced by 0.34 (0.62). Mean (SD) MetS-Z-BMI scores significantly differed depending on achievement of predetermined weight-based thresholds of ≥10% weight loss (patients aged ≥18 years) or ≥0.3-point reduction in BMI Z score (patients aged <18 years) at Week 52 (achievers, −0.64 [0.54]; nonachievers, 0.08 [0.47]; *P* = .0043). No significant difference was observed with other subgroup comparisons.

**Conclusion:**

MetS-Z-BMI score reductions were observed after 52 weeks of treatment, suggesting that setmelanotide may decrease metabolic syndrome severity and risk of future obesity-related comorbidities in those with BBS.

Bardet-Biedl syndrome (BBS) is a rare form of syndromic obesity resulting from variants in genes that disrupt the formation of the BBSome and subsequently impair primary ciliary formation and function (1-7). Currently variants in up to 27 genes have been associated with BBS (2-6, 8, 9). The primary features of BBS are rod-cone dystrophy, intellectual disability, obesity, polydactyly, and genital and renal abnormalities (1-6, 8-10). The clinical presentation of BBS varies between individuals, even within the same family (1). In addition to these clinical features, downstream impacts of impaired ciliary function can include alterations to leptin receptor trafficking to the cell surface (9, 11-13). Consequently, leptin signaling and the activation of the melanocortin-4 receptor (MC4R) pathway—a central regulator of hunger, food intake, and energy expenditure—can be impaired (2, 5, 9, 12, 13). Thus, variants in BBS-associated genes can result in attenuation of MC4R signaling, causing hyperphagia and early-onset, severe obesity, which are characteristically distressing symptoms experienced by patients with BBS (2, 5, 6). The obesity that occurs in patients with BBS is noteworthy because of its typical early onset, with studies showing ∼60% of patients developing obesity by the age of 5 years and ∼83% by 11 years (5). In adulthood, obesity in BBS is typically truncal, although it appears to be widespread and diffuse in childhood (10).

Hyperphagia and subsequent development of severe obesity in BBS can result in physiological changes consistent with metabolic syndrome and increase the likelihood of developing obesity-related comorbidities (1, 3-6, 8, 10, 14). Metabolic syndrome is a constellation of clinical features and is associated with a high risk of developing type 2 diabetes mellitus (T2DM) and cardiovascular disease (CVD) (15-19). Traditionally, metabolic syndrome has been confirmed with the presence of at least 3 abnormal components among adiposity measures (ie, waist circumference), blood pressure, fasting glucose, high-density lipoprotein (HDL) cholesterol, or triglycerides (15, 17). In comparison, a metabolic syndrome severity Z (MetS-Z) score, which is a continuous metric of metabolic syndrome severity, was developed using data from the National Health and Nutrition Examination Survey (1999-2010) and validated within a general population using data from the Atherosclerosis Risk in Communities and Jackson Heart Studies by DeBoer and Gurka et al (19-22). The calculation of MetS-Z scores incorporates factors to facilitate comparisons across groups of individuals, such as sex and race/ethnicity and age. MetS-Z scores can serve as an age-sensitive metric, as weight status can be incorporated either with body mass index (BMI) in both adult and pediatric patients or BMI Z scores in patients aged <18 years (MetS-Z-BMI score), or waist circumference in patients aged ≥18 years (19, 23, 24). DeBoer and Gurka et al have also reported that the odds for developing T2DM were increased by 2.7-fold in children and 2.8-fold in adults with every 1.0-point increase in MetS-Z-BMI score (15, 16, 19-21). This was also associated with an 9.8-fold increase in risk of developing CVD in children and a 2.4-fold increase in risk for adults (15, 16). Given that MetS-Z scores are continuous, they can also be used to monitor changes in severity over time, highlighting its clinical utility for identification of patients who may benefit from targeted early intervention as well as providing a method to track disease progression or response to treatment (3, 8, 15-17, 19, 20).

Although MetS-Z scores have been studied within the general population and in those who have general obesity (25, 26), to our knowledge, these investigations have not yet been extended to patients with hereditable conditions such as BBS and other MC4R pathway–related diseases of obesity. Recently, a phase 3 trial of the MC4R agonist setmelanotide demonstrated that 52 weeks of treatment resulted in reductions in weight-related measures and hunger in patients with BBS (2). Along with these results, metabolic parameters such as HDL cholesterol and triglycerides were improved after the 1-year treatment period. Given these findings, we examined metabolic syndrome severity at baseline and the effect of setmelanotide treatment on metabolic syndrome severity in patients with BBS-related obesity enrolled in this phase 3 trial.

## Methods

This secondary analysis used data from a phase 3 trial of patients with BBS who were treated with setmelanotide (NCT03746522) (2). Results from this trial have been previously published. Patients who had a confirmed diagnosis of BBS and had obesity, defined as BMI >97th percentile for patients aged 6 to <16 years or ≥30 kg/m^2^ for those aged ≥16 years were eligible for the trial. Patients were excluded from the study if they had moderate to severe renal dysfunction, undergone a diet or exercise program that resulted in ≥2% weight loss within the 2 months prior to screening, had sustained ≥10% weight loss from gastric bypass or obesity medications 3 months before screening, or had previous setmelanotide exposure. Patients were randomized to receive setmelanotide or placebo in a 14-week double-blind period, followed by an open-label treatment period in which all patients received setmelanotide for at least 52 weeks of treatment. Setmelanotide was titrated up to 3.0 mg over a 2-week period and continued at the therapeutic dose until Week 14. At Week 15, all patients transitioned to open-label setmelanotide at 3.0 mg until Week 66. Patients did not receive special guidance on lifestyle modifications related to dietary intake or physical activity during the trial, but patients aged <12 years received nutritional counseling to ensure appropriate development. This study protocol was approved by the institutional review board or independent ethics committee for each study site and the trial was undertaken in accordance with the International Council for Harmonisation Good Clinical Practice guidelines and the ethical principles founded in the Declaration of Helsinki. Written informed assent or consent was obtained from each participant or their parent or guardian.

Parameters assessed within the trial that were relevant to this analysis included change from baseline in body weight, waist circumference, lipid levels, and vital signs including blood pressure. Patients included in this analysis were those who had all data necessary to calculate MetS-Z-BMI score at baseline and Week 52, including weight and waist circumference, BMI (patients aged ≥18 years), BMI Z score (patients aged <18 years), fasting blood glucose, triglycerides, HDL cholesterol, and systolic blood pressure (SBP). Height data were imputed with baseline values for patients who did not have height data at Week 52. Patients’ history of disorders related to fasting glucose (diabetes, insulin resistance, impaired glucose tolerance, hyperinsulinemia), blood pressure (hypertension, arterial hypertension, increased blood pressure), and dyslipidemias were recorded. Treatment history and concomitant treatment during the study for any of these conditions were also recorded.

This analysis aimed to identify the change from baseline in MetS-Z-BMI scores following 52 weeks of setmelanotide treatment in this population of patients with BBS. For this purpose, MetS-Z-BMI scores were calculated using established age-, sex-, and race/ethnicity-specific equations at baseline and after Week 52 of treatment with setmelanotide (19, 21). Baseline values are the last recorded measurement before the first dose of setmelanotide (2). For MetS-Z-BMI score calculations, BMI served as the weight-based measure for patients aged ≥20 years, BMI Z scores were used for patients aged <20 years. BMI Z scores were calculated using a method established by the Centers for Disease Control and Prevention (24), in line with established sex- and race/ethnicity-specific equations (19, 21). The response in metabolic syndrome severity after treatment with setmelanotide was also assessed within the subgroups of sex, age, genotypic variant (*BBS1* vs *BBS10*), and weight-related response to setmelanotide treatment. Those who achieved a clinically meaningful weight-related response to treatment were classified as weight threshold achievers or nonachievers. For the purposes of this analysis, weight threshold achievers were defined as those who reached ≥10% weight loss (for patients aged ≥18 years) or a ≥ 0.3-point reduction in BMI Z score (for patients aged <18 years at baseline) after 1 year of active treatment. Additional assessments of subgroups were performed between weight threshold achievers and nonachievers for the variables of sex, age, and genotype (*BBS1* vs *BBS10*) where possible. Baseline MetS-Z-BMI scores were multiplied by 2.4 and 2.8, respectively, for adult patients and by 9.8 and 2.7, respectively, for pediatric patients to calculate a baseline odds ratio for developing CVD or T2DM (15, 16).

## Results

### Baseline Characteristics

Of the 32 patients with BBS enrolled and randomized, 22 were included in the current analysis (Table 1). Of the 10 patients excluded, 4 were excluded from the analysis due to missing data needed to calculate MetS-Z-BMI scores, 2 discontinued because of an adverse event, 2 were lost to follow-up, 1 withdrew, and 1 discontinued the study drug after receiving placebo but before receiving setmelanotide. Mean adherence to study drug was 91.4%. Nine patients were adults (age range, 20-44 years) at baseline; 13 were pediatric patients aged <18 years (age range, 10-16 years) at baseline. Variants in *BBS1* were the most common genetic background in the population (n = 10, 45.5%), followed by variants in *BBS10* (n =  9, 40.1%). The 10 excluded patients (6 who did not complete the trial and 4 who had missing data) had relatively varied genotypes and exhibited baseline characteristics similar to those of the patients included in this analysis.

**Table 1.** Demographics and baseline characteristics

Characteristic	Total (n = 22)	*BBS1* (n = 10)	*BBS10* (n = 9)	Other*^a^* (n = 3)
Age, mean (SD; range), y	20.3 (11.2; 10-44)	17.8 (8.0; 10-34)	21.4 (13.1; 12-43)	25.3 (16.7; 12-44)
Age, n (%)				
≥ 18 y	9 (40.9)	4 (40.0)	3 (33.3)	2 (66.7)
< 18 y	13 (59.1)	6 (60.0)	6 (66.7)	1 (33.3)
Sex, n (%)*^b^*				
Female	13 (59.1)	5 (50.0)	5 (55.6)	3 (100)
Male	9 (40.9)	5 (50.0)	4 (44.4)	0
Race, n (%)*^b^*				
White	20 (90.9)	9 (90.0)	8 (88.9)	3 (100)
Black or African American	1 (4.5)	0	1 (11.1)	0
Other	1 (4.5)	1 (10.0)	0	0
Ethnicity, n (%)*^b^*				
Not Hispanic/Latino	21 (95.5)	9 (90.0)	9 (100)	3 (100)
Hispanic/Latino	1 (4.5)	1 (10.0)	0	0
Weight, kg	112.9 (28.5)	113.4 (28.8)	116.6 (31.4)	100.4 (23.4)
BMI, kg/m^2,^ *^b^*	42.2 (9.9)	41.0 (10.0)	43.5 (11.5)	42.4 (5.1)
BMI Z score^*b*,*c*^	2.45 (0.42)	2.39 (0.51)	2.49 (0.40)	2.57 (NC)
Waist circumference, cm	118.2 (17.9)	120.7 (16.8)	116.8 (21.6)	113.8 (12.4)
SBP, mm Hg*^b^*	112.9 (9.4)	116.3 (9.1)	110.2 (10.1)	109.6 (6.8)
HDL cholesterol, mg/dL*^b^*	41.5 (6.3)	38.7 (5.8)	43.8 (6.7)	43.8 (4.4)
DBP, mm Hg	71.8 (10.5)	69.4 (11.0)	74.0 (11.3)	72.9 (6.4)
Triglycerides, mg/dL*^b^*	141.3 (67.1)	154.4 (58.4)	107.6 (44.4)	198.7 (114.7)
Fasting glucose, mg/dL*^b^*	86.4 (17.6)	80.9 (12.1)	88.1 (20.2)	99.7 (22.9)
ALT, U/L	38 (16)	48 (12)	31 (14)	24 (6)
AST, U/L	27 (7)	29 (7)	27 (8)	25 (3)
eGFR, mL/min/1.73 m^2^	101 (28)	101 (19)	109 (31)	79 (44)
MetS-Z-BMI score*^d^*	1.15 (0.54)	1.23 (0.33)	0.97 (0.60)	1.38 (0.93)
Odds ratio of developing CVD				
≥ 18 y	3.1 (1.4)	2.9 (0.8)	3.5 (1.2)	3.2 (2.2)
< 18 y	10.2 (4.5)	12.4 (3.0)	7.1 (4.2)	14.7 (NC)
Odds ratio of developing T2DM				
≥ 18 y	3.7 (1.6)	3.3 (0.9)	4.1 (1.3)	3.7 (2.6)
< 18 y	2.8 (1.3)	3.4 (0.8)	2.0 (1.1)	4.1 (NC)

Data are the mean (SD) unless noted otherwise. Normal values were based on American Board of Internal Medicine laboratory test reference ranges for ALT (10-440 U/L), AST (10-40 U/L), HDL cholesterol (>40-50 mg/dL), triglycerides (pediatric, < 90 mg/dL; adult, < 150 mg/dL), and fasting glucose (70-99 mg/dL), normal values reported by Centers for Disease Control and Prevention for SBP (<120 mg Hg) and BMI (18.5-24.9 kg/m^2^), BMI Z score (<1 SDS), and World Health Organization for BMI Z scores, reference values for eGFR are based on those provided by the National Kidney Foundation (normal function, ≥ 90 mL/min/1.73 m^2^; mild kidney impairment, 60-89 mL/min.

Abbreviations: ALT, alanine aminotransferase; AST, aspartate aminotransferase; BMI, body mass index; DBP, diastolic blood pressure; eGFR, estimated glomerular filtration rate; HDL, high-density lipoprotein; MetS-Z-BMI, metabolic syndrome Z score based on BMI; NC, not calculable; SBP, systolic blood pressure; SDS; standard deviation score; y, year.

^a^ *BBS2*, n = 2; *MKKS*, n = 1.

^b^Variable used in MetS-Z-BMI calculation (19, 21).

^c^BMI Z score reported for pediatric patients only (n = 13) using Centers for Disease Control and Prevention 2022 method.

^d^MetS-Z-BMI score calculations utilized BMI Z scores for patients who were aged ≥18 and ≤20 years.

With the exception of weight-related measures, triglycerides, and HDL cholesterol (in pediatric patients), the group mean for components used to calculate MetS-Z-BMI scores (ie, SBP, fasting glucose, HDL cholesterol [in adult patients]) were all within normal range. A full distribution of metabolic parameters across individual patients at baseline is included in Table 2. Two of 22 patients analyzed, one with *MKKS* and another with *BBS2* gene variants, had mild to moderate renal impairment (estimated glomerular filtration rate of 58 and 49 mL/min, respectively). Among the 22 patients, 11 had moderately high alanine aminotransferase (ALT) levels (*BBS1*, *BBS10*, and *MKKS*), 5 of whom also had elevated aspartate aminotransferase (AST) levels, but none of the elevations in either liver enzyme reached ≥3 times the upper limit of normal. Mean values and additional information regarding mean change from baseline in metabolic parameters and concomitant therapies within this patient population can be found in Table 3. The mean (SD) MetS-Z-BMI score at baseline across all patients was 1.15 (0.54). At baseline, adults had a mean (SD) odds ratio of developing CVD or T2DM of 3.1 (1.5) and 3.7 (1.7), respectively. Pediatric patients at baseline had a mean (SD) odds ratio for developing CVD or T2DM of 10.2 (4.7) and 2.8 (1.3), respectively. Calculated odds ratios are listed by individual in Table 4.

**Table 2.** Baseline and week 52 metabolic parameters in individual patients

Baseline									Change from baseline at Week 52			
Patient	Age, y	Sex	Gene	Variant details	Systolic blood pressure, mm Hg	Triglycerides, mg/dL	HDL cholesterol, mg/dL	Fasting glucose, mg/dL	Systolic blood pressure, mm Hg	Triglycerides, mg/dL	HDL cholesterol, mg/dL	Fasting glucose, mg/dL
1	43	F	*BBS10*	CHET-PLP/PLP	103.3*^a,b^*	184.2	46.4	135.1*^a,b^*	0.4*^b^*	−75.3	0.0	−57.6*^b,c^*
2	13	M	*BBS1*	HOM-PLP/PLP	129.0	120.5	30.9	99.1	−7.3	−84.2	3.9	−5.4
3	42	F	*BBS10*	CHET-PLP/PLP	116.7*^a,b^*	143.5	50.3	97.3	−0.4*^b^*	−41.6	7.7	−14.4
4	12	F	*MKKS*	HOM-VUS(SP)/VUS(SP)	115.0*^a,b^*	269.3	46.4	86.5*^a,b^*	−14.3*^b^*	−125.8	7.7	−5.4*^b^*
5	20	F	*BBS2*	CHET-PLP/PLP	111.7	66.4	46.4	86.5	0.6	−17.7	11.6	−1.8
6	34	M	*BBS1*	HOM-PLP/PLP	117.7*^a,b^*	124.0	30.9*^a^*	93.7	8.3*^b^*	−41.6	3.9	−7.2
7	29	M	*BBS10*	HOM-PLP/PLP	107.7	135.5	42.5	88.3	18.3	−25.7	3.9	−12.6
8	15	F	*BBS10*	HOM-PLP/PLP	104.7	121.3	42.5	81.1	−9.7	−3.5	7.8	0.0
9	12	M	*BBS10*	CHET-PLP/PLP	120.7	82.4	54.1	91.9	−26.4	1.7	−7.7	−7.2
10	20	M	*BBS1*	CHET-PLP/VUS(SP)	118.3*^a,b^*	258.6	42.5	73.9*^a,b^*	−18.6*^b,c^*	−94.7	3.9	−5.4*^b^*
11	12	F	*BBS10*	CHET-PLP/PLP	98.7	116.0	42.5	77.5	10.6	8.0	3.9	3.6
12	13	F	*BBS1*	HOM-PLP/PLP	119.3	99.2	34.8	54.0*^a,b^*	−20.0	49.6	−3.9	14.5*^b^*
13	21	F	*BBS1*	HOM-PLP/VUS	114.3	164.7	50.3	84.7	−9.3	110.8	15.4	0.0
14	28	F	*BBS1*	HOM-PLP/PLP	119.0*^a,b^*	170.1	38.7	77.5*^a,b^*	8.3*^b^*	−46.1	0.0	5.4*^b^*
15	12	M	*BBS10*	HOM-PLP/PLP	128.0	78.8	38.7	75.7	−6.3	6.2	0.0	−3.6
16	14	F	*BBS10*	CHET-PLP/VUS/VUS	113.3	62.9	30.9	63.1	−9.0	−14.2	0.0	18.0
17	44	F	*BBS2*	CHET-PLP/PLP	102.0*^a,b^*	260.4	38.7	126.1*^a,b^*	1.3*^b^*	−47.8	−3.9*^b^*	16.2*^b,c^*
18	14	M	*BBS10*	CHET-PLP/PLP	99.0	43.4	46.4	82.9	20.0	8.9	0.0	−3.6
19	10	M	*BBS1*	HOM-PLP/PLP	105.3	205.5	38.7*^a,b^*	77.5*^a,b^*	10.0	73.5	0.0	1.8*^b^*
20	10	F	*BBS1*	HOM-PLP/PLP	107.7	85.0	42.5	82.9	16.6	8.9	3.9	3.6
21	16	M	*BBS1*	HOM-PLP/PLP	129.7	217.0	38.7	81.1	44.0	−91.2	−3.9	3.6
22	13	F	*BBS1*	CHET-PLP/PLP	103.0	99.2	38.7	84.7	32.3	−2.7	0.0	5.4

Abbreviations: CHET, compound heterozygous; HDL, high-density lipoprotein; HOM, homozygous; PLP, predicted likely pathogenic; SP, suspected pathogenic; VUS, variant of uncertain significance.

^a^Patient had a medical history of dysfunction related to blood pressure (hypertension), HDL cholesterol (dyslipidemia), or fasting glucose (type 2 diabetes, hyperinsulinism, insulin resistance, or impaired glucose tolerance).

^b^Patient was receiving treatment for a dysfunction related to measurement at baseline that continued through the study.

^c^Patient initiated treatment for a dysfunction related to measurement during the trial.

**Table 3.** Change in metabolic parameters used in MetS-Z-BMI calculation and change in MetS-Z-BMI score at week 52*^a^*

Mean (SD)	Week 52 (N = 22)	Change from baseline (N = 22)
BMI, kg/m^2^	38.2 (10.1)	−4.1 (3.0)
BMI Z score***^b^***	2.10 (0.75)	−0.35 (0.37)
Systolic blood pressure, mm Hg	115.2 (17.6)	2.2 (17.2)
HDL cholesterol, mg/dL	43.9 (9.4)	2.5 (5.4)
Triglycerides, mg/dL	121.1 (65.0)	−20.2 (56.2)
Fasting glucose, mg/dL	84.0 (14.4)	−2.4 (14.9)
MetS-Z-BMI score	0.80 (0.71)	−0.34 (0.62)

Abbreviations: BMI, body mass index; HDL, high-density lipoprotein; MetS-Z-BMI, metabolic syndrome severity based on body mass index.

^a^Common concomitant medications taken during the study included vitamin D and analogues, angiotensin-converting enzyme inhibitors, anilides, biguanides, osmotically acting laxatives, progestogens and estrogens, and selective beta-2-adrenoreceptor agonists.

^b^BMI Z score calculated for pediatric patients aged <18 years (n = 13).

**Table 4.** Odds ratio at baseline*^a^* of developing future CVD or T2DM

Patient	Age, y	Sex	Gene	CVD odds ratio	T2DM odds ratio
1	43	F	*BBS10*	4.6	5.4
2	13	M	*BBS1*	14.9	4.1
3	42	F	*BBS10*	4.0	4.7
4	12	F	*MKKS*	14.7	4.1
5	20	F	*BBS2*	1.0	1.1
6	34	M	*BBS1*	2.2	2.5
7	29	M	*BBS10*	1.9	2.3
8	15	F	*BBS10*	10.3	2.8
9	12	M	*BBS10*	5.9	1.6
10	20	M	*BBS1*	3.7	4.4
11	12	F	*BBS10*	4.8	1.3
12	13	F	*BBS1*	12.0	3.3
13	21	F	*BBS1*	3.5	4.1
14	28	F	*BBS1*	2.0	2.3
15	12	M	*BBS10*	10.9	3.0
16	14	F	*BBS10*	11.3	3.1
17	44	F	*BBS2*	5.4	6.3
18	14	M	*BBS10*	−0.5	−0.1
19	10	M	*BBS1*	12.3	3.4
20	10	F	*BBS1*	11.1	3.1
21	16	M	*BBS1*	16.9	4.7
22	13	F	*BBS1*	7.4	2.0

Abbreviations: CVD, cardiovascular disease; T2DM, type 2 diabetes mellitus.

^a^Each 1.0-point in MetS-Z-BMI score during childhood and adulthood increases the odds of future CVD by 9.8 and 2.4, respectively, and for T2DM by 2.7 and 2.8.

### MetS-Z-BMI Score at Week 52

**Figure 1.**
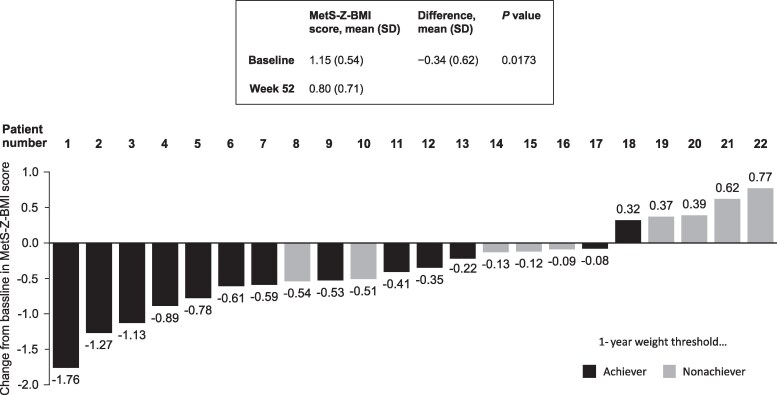
Distribution of MetS-Z-BMI score change from baseline at Week 52. MetS-Z-BMI, metabolic syndrome severity based on body mass index.

**Figure 2.**
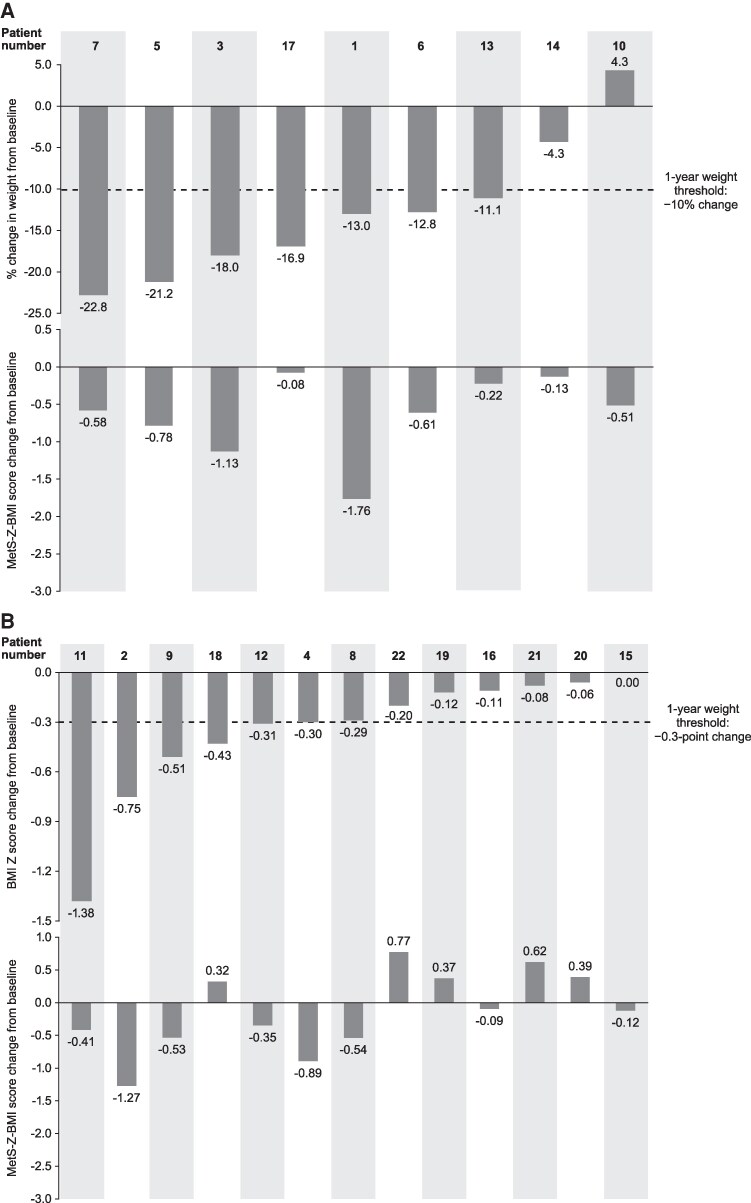
Change from baseline based on weight response with setmelanotide treatment. **(**A**)** Percent change in weight (top) and change in MetS-Z-BMI score (bottom) in adult patients. **(**B**)** Change in BMI Z score (top) and MetS-Z-BMI score (bottom) for pediatric patients. The same individual is aligned across top and bottom parts of each panel representing the distribution of individual change from baseline at Week 52. MetS-Z-BMI, metabolic syndrome severity based on body mass index.

Decreases were observed in the overall group mean of all metabolic parameters at Week 52, with the exception of SBP, which remained in the normal range from baseline (Table 3). This was also observed in the pediatric and adult subgroups, with the exception of mean fasting glucose in pediatric patients, which remained in the normal range from baseline. Individual changes in SBP, triglycerides, HDL cholesterol, and fasting glucose to Week 52 are shown in Table 2. Overall, mean (SD) change from baseline to Week 52 in MetS-Z-BMI score was −0.34 (0.62; *P *= .0173), ranging from −1.76 to 0.77 (Fig. 1; Table 3). The range in MetS-Z-BMI score change was −1.76 to −0.08 in adult patients and −1.27 to 0.77 in pediatric patients. Of the 22 patients, 13 (59.1%) were classified as achieving the 1-year weight-reduction threshold (≥10% weight loss in patients aged ≥18 years, or ≥0.3-point decrease in BMI Z score in patients aged <18 years); however, 17 of 22 patients (77.2%) showed a reduction in MetS-Z-BMI score at Week 52, including 5 nonachievers of the 1-year weight threshold (Fig. 2; Table 5).

**Table 5.** Baseline and weight and MetS-Z-BMI score in individual patients

Patient characteristic				Baseline		Change from baseline at Week 52	
Patient number	Age, years	Sex	Gene	BMI or BMI Z score*^a^*	MetS-Z-BMI score	BMI or BMI Z score*^a^*	MetS-Z-BMI score
1	43	F	*BBS10*	43.0	1.92	−4.7	−1.76
2	13	M	*BBS1*	1.52	1.52	−0.75	−1.27
3	42	F	*BBS10*	54.8	1.66	−9.8	−1.13
4	12	F	*MKKS*	2.57	1.50	−0.30	−0.89
5	20	F	*BBS2*	43.80	0.40	−7.7	−0.78
6	34	M	*BBS1*	39.4	0.90	−4.5	−0.61
7	29	M	*BBS10*	47.4	0.81	−8.4	−0.59
8	15	F	*BBS10*	2.65	1.05	−0.29	−0.54
9	12	M	*BBS10*	2.14	0.60	−0.51	−0.53
10	20	M	*BBS1*	57.8	1.55	3.0	−0.51
11	12	F	*BBS10*	1.91	0.49	−1.38	−0.41
12	13	F	*BBS1*	2.56	1.23	−0.31	−0.35
13	21	F	*BBS1*	54.6	1.45	−4.8	−0.22
14	28	F	*BBS1*	44.9	0.82	−1.4	−0.13
15	12	M	*BBS10*	2.96	1.11	0.00	−0.12
16	14	F	*BBS10*	2.82	1.15	−0.11	−0.09
17	44	F	*BBS2*	46.8	2.25	−6.8	−0.08
18	14	M	*BBS10*	2.45	−0.05	−0.43	0.32
19	10	M	*BBS1*	2.80	1.26	−0.12	0.37
20	10	F	*BBS1*	2.87	1.13	−0.06	0.39
21	16	M	*BBS1*	2.52	1.73	−0.08	0.62
22	13	F	*BBS1*	2.07	0.76	−0.20	0.77

Abbreviations: BMI, body mass index; BBS, Bardet-Biedl Syndrome; MetS-Z-BMI, metabolic syndrome Z score based on BMI.

^a^BMI (kg/m^2^) is reported for patients ≥18 years; BMI Z score is reported for patients <18 years.

**Figure 3.**
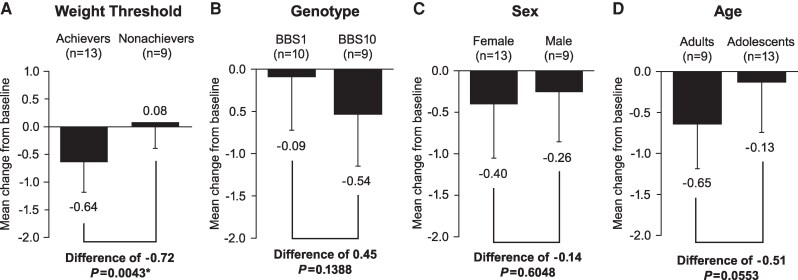
Comparison of mean Met-Z-BMI score at baseline and Week 52 within subgroups of **(**A**)** 1-year weight threshold, **(**B**)** genotype, **(**C**)** sex, and **(**D**)** age. *Significant difference between groups. Absolute difference is reported for each subgroup analysis. Error bars are the SD. *P* values were calculated using a 2-sided 2-sample *t* test. Analysis of genotype did not include patients with *BBS2* (n = 2) or *MKKS* (n = 1).

Patients achieving the predetermined weight loss threshold at 1 year had significantly greater change in mean MetS-Z-BMI score compared with nonachievers (Fig. 3; Table 6). The mean (SD) change from baseline in MetS-Z-BMI score was −0.64 (0.54) for 1-year weight threshold achievers and 0.08 (0.47) for nonachievers (*P *= .0043). No significant differences were observed at Week 52 within the subgroups of sex, age, or genotype (Fig. 3); further, when comparing the sex, age, or genotype subgroup within subgroups of weight threshold achievement, no significant differences were observed (Table 6).

**Table 6.** Assessment of MetS-Z-BMI score changes by achievement of 1-year weight threshold and demographic subgroup and disease characteristics

1-Year weight threshold group	n	Achiever	n	Nonachiever
**Overall, mean (SD)**	13	−0.64 (0.54)	9	0.08 (0.47)
Difference, achiever − nonachiever	−0.72, *P* = 0.0043			
**Genotype, mean (SD)**				
* BBS1*	4	−0.61 (0.47)	6	0.25 (0.52)
* BBS10*	6	−0.68 (0.70)	3	−0.25 (0.25)
Difference, *BBS10—BBS1*		0.07, *P* = 0.866		0.33, *P* = 0.142
**Sex, mean (SD)**				
Female	8	−0.70 (0.56)	5	0.08 (0.51)
Male	5	−0.54 (0.57)	4	0.09 (0.51)
Difference, female − male		0.17, *P* = 0.609		0.01, *P* = 0.980
**Age, mean (SD)**				
Adults	7	−0.74 (0.57)	2	−0.32 (0.27)
Pediatric	6	−0.52 (0.54)	7	0.20 (0.47)
Difference, adults − pediatric		−0.22, *P* = 0.500		−0.52, *P* = 0.185

*P* values were calculated using a 2-sided 2-sample unpaired *t* test. Weight threshold achiever is defined as weight loss ≥10% in patients aged ≥18 years or ≥0.3-point change in BMI Z score in patients aged <18 years after 1 year of treatment. Abbreviation: MetS-Z-BMI, metabolic syndrome severity based on body mass index.

## Discussion

Individuals from this cohort of patients with BBS exhibited a mean MetS-Z-BMI score (adult, 1.3; pediatric, 1.0) consistent with that observed in those with general and early-onset obesity (25, 26). Because any increase in MetS-Z score has been associated with an increased likelihood for developing CVD and T2DM in pediatric and adult patients with general obesity (15, 16, 21), this assessment of metabolic syndrome severity in patients with BBS suggests patients with this condition are also at an increased risk for development of CVD and T2DM (8, 15-17, 20). Additionally, treatment with setmelanotide resulted in a mean reduction in MetS-Z-BMI scores after 1 year of treatment, indicating that setmelanotide has the potential to reduce the severity of metabolic syndrome, in addition to weight and hunger in patients with BBS (2).

Generally, patients with the greatest decreases in MetS-Z-BMI scores were those who achieved a predetermined weight threshold after 1 year of treatment. The correlation of change in MetS-Z-BMI scores and change in weight-based parameters highlights the utility of this metric and suggests it may hold potential for assessment of intervention benefit. While changes to weight-related measures are the traditional assessment of efficacy with anti-obesity therapies (27, 28), efficacy focused on achievement of weight-based thresholds may obscure benefits in patients who do not meet these endpoints, despite experiencing reductions or maintenance of weight with treatment. While some patients demonstrated reductions in MetS-Z-BMI scores that were generally associated with weight-related reductions, similar associations between MetS-Z-BMI scores and weight-related reductions were not observed in 5 patients who did not show a robust decrease in weight (range for BMI Z score decrease in 3 pediatric patients, −0.29 to 0; range for percent change in weight in 2 adult patients, −4.3 to 4.3). However, these patients still exhibit a decrease in their respective MetS-Z-BMI score (range, −0.54 to −0.09). These results indicate MetS-Z-BMI scores may provide an additional indication of clinical benefit beyond change in weight-related measures (29). This highlights the potential of MetS-Z-BMI scores to serve as an additional metric of treatment benefit, as these scores encompass parameters of fasting glucose, blood pressure, HDL cholesterol, and triglycerides (19, 21, 23). Utilizing MetS-Z scores as a supplemental metric in clinical studies may identify patients who receive benefits of some weight loss or improvement in metabolic parameters despite not achieving predefined weight-related measures of efficacy (15-17, 19, 20).

While using MetS-Z scores as a metric of efficacy can allow for improved assessment of disease severity and response to treatment, it may not be sufficient to identify a response across all individuals. One reason for this may be the state of patients at baseline. For example, patients with high metabolic syndrome severity at baseline who receive benefit from treatment have the potential for a much greater change with treatment compared with patients who have low metabolic syndrome severity at baseline who may already be at or close to the normal range for the components used to calculate MetS-Z scores. Additionally, given that a reduction in weight is shown to improve abnormal components of MetS-Z scores (ie, reduced fasting glucose and blood pressure and increased HDL cholesterol), those who maintain weight over the duration of the trial may not exhibit as great a change in MetS-Z scores with treatment as patients who experienced significant weight loss (30, 31). Further, patients who maintain weight loss over the course of the trial may experience a change in weight gain trajectory as a result of treatment (ie, weight gain may have continued in the absence of treatment). In such cases, MetS-Z scores cannot accurately reflect the benefit of preventing increased metabolic syndrome severity over time. The mixed response to setmelanotide treatment across both MetS-Z scores and weight-related parameters in this population of patients with BBS indicate the general need for a more robust examination of changes across individuals. Although MetS-Z scores can provide an expanded and detailed assessment of treatment response as identified through changes across the relevant parameters (19, 21, 23), the primary focus of obesity pharmacotherapies is weight management (2, 27, 28). Thus, the variability of change in both MetS-Z-BMI scores and weight-related parameters in individuals within this population highlight that MetS-Z scores should be used to supplement rather than replace existing standards that determine treatment efficacy (ie, changes in weight-related parameters).

A difference in treatment efficacy may also be obscured by the characteristically diverse clinical presentation in patients with BBS and individual histories of weight trajectory (1, 3, 5, 6, 10, 14, 32). Differences in clinical presentation and response to treatment among patients with BBS can also be attributed to the diversity of causative genetic variants (1, 3, 5, 6, 9, 14). BBS genotypes typically cluster into those that encode BBSome proteins (*BBS1*, *BBS2*, *BBS4*, *BBS5*, *BBS7*, *BBS8*, *BBS9*, *BBS18*) and those that encode chaperonin proteins (*BBS6*, *BBS10*, *BBS12*) (3, 5, 6, 8-10, 14). The most common variants among patients with BBS in Europe and North America are those in *BBS1* and *BBS10*, consistent with the population of patients used in this analysis (1, 4, 14). Comparisons of patients with variants in *BBS1* vs *BBS10* have found *BBS10* pathogenic variants to result in an increased disease severity as identified through increased incidence and risk for kidney disease, metabolic syndrome, CVD, and severity of childhood obesity (1, 3-6). Within this small population of patients with BBS, no significant difference was observed in MetS-Z-BMI scores between *BBS1* and *BBS10* either at baseline or after 1 year of setmelanotide treatment. However, while not significant, patients with variants in *BBS10* did have a numerically greater reduction in MetS-Z-BMI scores after setmelanotide treatment compared with patients with variants in *BBS1* (−0.54 and −0.09, respectively), which was also aligned with change in weight across pediatric (BMI Z score reduction, −0.45 and −0.25, respectively) and adult (mean percent change in BMI, −16.4 and −6.2, respectively) patients. It is possible that the small sample size and combined pediatric and adult data may have confounded differences between patients with different variants and that those with variants in *BBS10* may have achieved a greater reduction over that observed with a less severe presentation in those with variants in *BBS1* (1, 8). However, further investigation with a larger sample size is needed for an accurate assessment of the influence of genotypic differences on the response of metabolic syndrome severity in patients with BBS treated with setmelanotide.

While the study presented here underscores the application of MetS-Z-BMI scores for assessment of patients with BBS, there are a few limitations. Primarily, the MetS-Z score and associated risk for T2DM and CVD was developed and validated in the general population and not in the syndromic patient population studied here. However, in the absence of a similar measurement designed for patients with BBS, we felt this was a valid hypothesis generating approach. Also, as mentioned previously, the small sample size limited the analyses between various subgroups, and combined adult and pediatric data may be a confounding variable. A combined analysis of adult and pediatric patients can impact the effect of treatment given the ongoing change in body composition of pediatric patients. Age can further influence the impact of treatment, as the severity of underlying disease (BBS) and the severity of some components of metabolic syndrome, such as obesity, differ between adults and pediatric patients (1, 5). Another limitation of this study is that some included patients had preexisting conditions and were receiving concomitant medications that were related to and could affect the risk of developing CVD or T2DM. However, because these preexisting conditions were not part of the exclusion criteria for the original phase 3 trial, patients with these conditions were also not excluded from the current analyses. As previously noted, although kidney disease and liver enzyme elevations are common comorbidities in patients with BBS and could impact CVD risk, in this small study group, there were no patients with severe kidney disease or abnormal liver enzymes ≥3 times the upper limit of normal at baseline or at Week 52 and there was no difference identified in these values when comparing individuals with variants in *BBS1* or *BBS10* (1, 33, 34). Thus, while these results may not be reflective of what might be observed in patients with renal or hepatic impairments, it is unlikely that CVD risk in this patient population was impacted by change in kidney or liver function. Further, changes to underlying components other than obesity (ie, glucose, triglycerides, HDL cholesterol, and blood pressure) can also result in a corresponding change to individual MetS-Z scores, which underscores the utility of this metric as a complementary assessment of treatment efficacy rather than a replacement to traditional weight-related endpoints (19, 21, 23). Additionally, equations used for the calculation of MetS-Z scores were derived from a US population (15, 16, 23). The patients evaluated in this study comprise patients with BBS who are predominantly of European descent. It is possible that adjustments to cofactors used for MetS-Z-BMI score calculations may be necessary for the most accurate assessment of metabolic syndrome severity in this patient population or in general when comparing individuals across geographic regions or cultures. Regardless of these limitations, changes to MetS-Z-BMI scores in this population of patients with BBS generally are associated with changes in typical weight-related measures used to assess efficacy with setmelanotide. It is also important to note that although studies have investigated and suggested a minimally important clinical difference for change in metabolic syndrome severity scores, a verified standard threshold for a clinically meaningful improvement of metabolic syndrome severity has not yet been established (15, 16, 18, 20). Despite this outstanding need, metabolic syndrome severity scores maintain their utility through their continuous nature, enabling the monitoring of patients over time and providing physicians with ongoing information to inform treatment strategies (15, 16, 18, 20, 23).

In conclusion, assessments of MetS-Z-BMI scores demonstrate metabolic syndrome severity and its response to treatment after 1 year of setmelanotide in this population of patients with BBS. Setmelanotide treatment was associated with a significant reduction in MetS-Z-BMI scores between baseline and Week 52 for both adult and pediatric patients with BBS, and those who met weight-reduction thresholds exhibited a greater reduction in MetS-Z-BMI scores compared with those who did not. Additionally, although 8 patients did not achieve weight-based thresholds, 5 of these patients did exhibit a reduction in MetS-Z-BMI scores after setmelanotide treatment. Further, changes in MetS-Z-BMI scores suggest setmelanotide treatment may lessen metabolic syndrome severity, even in the absence of significant weight reduction. These findings support further investigation into the impact of setmelanotide on the risk for obesity-related comorbidities, which may be achieved with future development of MetS-Z scores to allow for the extrapolation of change in metabolic syndrome severity with a change in risk of CVD and T2DM.

## Data Availability

Restrictions apply to the availability of some or all data generated or analyzed during this study to preserve patient confidentiality or because they were used under license. The corresponding author will on request detail the restrictions and any conditions under which access to some data may be provided.

## Clinical Trial Identifier

NCT03746522

## Abbreviations

BBS: Bardet-Biedl syndrome
BMI: body mass index
CVD: cardiovascular disease
HDL: high-density lipoprotein
MC4R: melanocortin-4 receptor
MetS-Z score: metabolic syndrome severity Z score
SBP: systolic blood pressure
T2DM: type 2 diabetes mellitus
